# MicroRNA‐1258, regulated by c‐Myb, inhibits growth and epithelial‐to‐mesenchymal transition phenotype via targeting SP1 in oral squamous cell carcinoma

**DOI:** 10.1111/jcmm.14189

**Published:** 2019-02-07

**Authors:** Hua Zhang, Sui Jiang, Longbin Guo, Xi Li

**Affiliations:** ^1^ Department of Medical Oncology Guangdong General Hospital and Guangdong Academy of Medical Sciences Guangzhou China; ^2^ Department of Oral and Maxillofacial Surgery Guangdong General Hospital and Guangdong Academy of Medical Sciences Guangzhou China; ^3^ Department of Operation Room Affiliated Cancer Hospital of Guangzhou Medical University Guangzhou China; ^4^ Department of Thyroid and Breast Surgery The Third Affiliated Hospital of Sun Yat‐Sen University Guangzhou China

**Keywords:** c‐Myb, miR‐1258, oral squamous cell carcinoma, SP1

## Abstract

The biological function and underlying mechanism of miR‐1258 has seldom been investigated in cancer progression, including in oral squamous cell carcinoma (OSCC). In the current study, we revealed that the expression level of miR‐1258 was significantly down‐regulated in OSCC tissues and cell lines. Restoration of miR‐1258 decreased OSCC cell growth and invasion. The luciferase and Western blot assays revealed that SP1 protein was a downstream target of miR‐1258. Overexpression of SP1 dismissed miR‐1258’s effect on cell growth and invasion. We also revealed that c‐Myb inhibited miR‐1258 by directly binding at its promoter. In addition, miR‐1258 inhibited PI3K/AKT and ERK signalling pathway activity. Taken together, these findings demonstrated that miR‐1258 may function as a tumour‐suppressive micorRNA in OSCC and suggested that miR‐1258 may be a potential therapeutic target for OSCC patients.

**Abbreviations**
3'‐UTRs: 3'‐untranslated regions (UTRs)
CHIP: chromatin immunoprecipitation
EMT, epithelial‐to‐mesenchymal transition phenotype
miR: microRNA
OSCC: oral squamous cell carcinoma
TFBSs: transcription factor‐binding sites

## 1 INTRODUCTION

Ranking the sixth most common cancer worldwide, oral squamous cell carcinoma (OSCC) accounts for >40% of head and neck malignancies.^1^ The OSCC often displays high rates of lymph node metastasis when diagnosed, which contributes to poor prognosis of the disease. Approximately up to 50% of OSCC patients present with advanced disease and metastasis.^2^ Therefore, it is urgent to clarify the molecular pathogenesis underlying the aggressive progression of OSCC.

As a small and single‐stranded RNAs, microRNAs (miRNAs) bind at the 3'‐untranslated regions (UTRs) of target genes and negatively regulate the downstream gene expression. The role of miRNAs in regulating cancer progression has been fully investigated, including in OSCC.^3^, ^4^ The function of miR‐1258 has been reported in gastric cancer, liver cancer and breast cancer.^5^, ^6^, ^7^, ^8^ MiR‐1258 was down‐regulated in colorectal cancer (CRC) tissues and CRC cell lines, and up‐regulated miR‐1258 was proved to inhibit proliferation and arrest cell cycle at G0/G1 in vitro and vivo.^9^ MiR‐1258 suppresses tumour progression via GRB2/Ras/Erk pathway in non‐small‐cell lung cancer.^10^ These literatures suggested that miR‐1258 may function as a tumour suppressor. However, the underlying role of miR‐1258 in human OSCC is still poorly understood.

We revealed that miR‐1258 was down‐regulated in OSCC tissues and cell lines. We also demonstrated that miR‐1258 decreased growth and invasion in OSCC via targeting the SP‐1 protein. In particular, we demonstrated that c‐Myb decreased miR‐1258 expression in OSCC by biding to its promoter. Targeting c‐Myb/miR‐1258/SP‐1 axis may be a promised strategy for OSCC.

## 2 MATERIALS AND METHODS

### 2.1 OSCC cell lines culture, cell transfection and OSCC patient samples

The two OSCC cell lines SCC‐9 and SCC‐15 were obtained from the Institute of Chemistry and Cell Biology of the Chinese Academy of Sciences (Shanghai, China). They were cultured in DMEM medium supplemented with 10% foetal bovine serum.

For transfection, cells were cultured to 80% confluence and transfected with miR‐1258, miR‐1258 inhibitor or miR‐ctrl using Lipofectamine 2000 (Invitrogen) according to the manufacturer's recommendation.

The OSCC patient samples were collected from Guangdong General Hospital. All the experiments were performed in accordance with the approved guidelines of the Institutional Research Ethics Committee of the Guangdong General Hospital.

### 2.2 Real‐time PCR assay

Total RNA were extracted from OSCC cells and tissues by using TRIzol reagent (Invitrogen). Detection of miR‐1258 levels was carried out by using the qRT‐PCR miRNA kit (Ruibo, Guangzhou, China). Under the ABI PRISM 7500 Sequence Detection System (ABI), the real‐time PCR assay was performed by using a SYBR Green kit (TaKaRa, Tokyo, Japan). MiR‐1258 forward primer: (5′‐CTGCGAGTCCCTGGAGTTAG‐3′), reverse primer (5′‐CGGTCCCCTA‐ACTACCCATT‐3′); U6 forward primer: (5′‐ATACAGAGAAAGTTAGCACGG′), reverse primer (5′‐GGAATGCTTCAAAGAGTTGTG′); GAPDH forward primer: (5′‐TCAAGATCATCAGCAATGCC′), reverse primer (5′‐ CGATACCAAAGTTGTCATGGA′); SP‐1 forward primer: (5′‐GTCATACTGTGGGAAACG′), reverse primer (5′‐GCAAATTTCTTCTCACCTGTG′).

### 2.3 Production of lentivirus and cell infection

The plasmid carrying miR‐1258 (Cat.NO. mh10085) and the control plasmid (Cat.NO. m001) were purchased from Applied Biological Materials (ABM) Inc We named this vector LV‐miR‐1258 and the control plasmid was named LV‐ctrl. The package of viruses was carried out as the standard protocol. About 72 hours later, the virus particles were harvested and stored in −80°C. Subsequently, cells were infected with virus particles and 8 μg/ml polybrene.

### 2.4 Colony formation and MTT assay

To perform colony formation assay, 200 cells were seeded in six‐well culture plate and cultured for 2 weeks. After that, we washed the cells three times with PBS and stained them with Giemsa solution. Subsequently, the number of colonies containing ≥50 cells was counted under a microscope. The plate clone formation efficiency was evaluated by using the formula:  = (number of colonies/number of cells inoculated) × 100%.

The MTT assay was carried out as previously described.^11^ Briefly, cells were seeded on 96‐well plate and were allowed to grow for 24 hours. The media were aspirated and MTT solution was added into each well. After incubation for 30 minutes, 150 µL DMSO was added into each well. Finally, the absorbance was read at OD = 590 nm.

### 2.5 Cell invasion ability assay

The cell invasion ability was examined by Boyden assay. The cells were seeded into the upper chambers (Millipore) that are coated with 150 µg Matrigel (BD Biosciences, Boston, MA, USA). Below the upper chambers were the lower chambers that were filled with 500 µL DMEM supplemented with 10% FBS. After incubation for 12 hours, the cells adhering to the lower surface were fixed with methanol, stained with Giemsa solution and counted.

### 2.6 Western blot assay

The total proteins were extracted from cells with RIPA buffer (Beyotime, China) and were then separated by SDS‐PAGE gel, followed by transfer to polyvinylidene fluoride (PVDF) membranes. We blocked the membranes by using 3% BSA/TBST and incubated them with primary antibodies at 4°C overnight. We then rinsed the PVDF membranes three times for 5 minutes with TBST and incubated them in HRP‐conjugated secondary antibodies for 1 hour at room temperature. We detected the levels of total protein were by using enhanced chemiluminescence reagents. The primary antibodies SP‐1 (Lot. No. ab13370), cyclind1 (Lot. No. ab134175), CDK4 (Lot. No. ab108357) and GAPDH (Lot. No. ab9485) were purchased from Abcam. The concentration of the antibodies used in the study was 1:500. The primary antibodies E‐cadherin (Lot. No. sc‐71009), N‐cadherin (Lot. No. sc‐53488) and Vimentin (Lot. No. sc‐73258) were purchased from Santa Cruz. The concentration of the antibodies used in the study was 1:500.

### 2.7 Luciferase reporter assay

We cloned the full‐length SP‐1 cDNA (lacking the 3′‐UTR) into the eukaryotic expression vector pcDNA3.1 (Invitrogen). Subsequently, the 3'‐UTR untranslated region of SP‐1 was amplified and cloned downstream of the firefly luciferase gene in the pGL3 vector (Promega) and the vector as named wild‐type (WT) SP‐1‐3'‐UTR. By using GeneTailor Site‐Directed Mutagenesis System (Invitrogen), we made site‐directed mutagenesis of the miR‐1258 biding sites in the SP‐1 3'‐UTR. The vector was named mutant type (MUT) SP‐1‐3'‐UTR. Subsequently, we cotransfected the OSCC cells with the wt or mut SP‐1‐3’UTR vector and miR‐1258 mimic or inhibitor. Finally, we performed the luciferase assay by using the dual Luciferase reporter assay system (Promega) 36 hours after transfection. To perform miR‐1258 promoter luciferase assays, OSCC cells were seeded into 24‐well plates and cotransfected with plasmids that contains miR‐1258 promoter and the pRL‐TK‐Renilla plasmid (Promega, USA).

### 2.8 Chromatin immunoprecipitation (CHIP) assay

We performed the CHIP assay according to the manufacturer's instructions by a ChIP assay kit (Millipore, catalog: 17‐371). Briefly, the cells were fixed with 1% formaldehyde to covalently crosslink proteins to DNA followed by harvesting chromatin from the cells. Subsequently, the crosslinked DNA (sheared to 200‐1000 base pairs in length) that linked with sonication were processed to an immmmunoselection process. Then the PCR assay was performed to measure enrichment of DNA fragments in the putative c‐Myb‐binding sites in the miR‐1258 promoter.

### 2.9 Tumour xenograft experiments

The female BALB/c nude mice (5‐week‐old) were fed under standard conditions and cared according to the institutional guidelines for animal care. The animal experiments were approved by the Institutional Animal Care and Use Committee of Guangdong General Hospital. The LV‐miR‐1258 and LV‐miR‐ctrl cells were injected subcutaneously into the posterior flank of the mice. We calculated tumour volumes by using the formula (volume = length × width2/2). Five weeks after the implantation, the xenografts were removed from the mice and the xenografts were weighed. Then the Ki‐67 stain assay was carried out to evaluate the proliferation index.

## 3 RESULTS

### 3.1 The expression of miR‐1258 in OSCC tissues

First, we used TGCA database to find out the abnormal expression of microRNAs in head and neck cancer (HNSC). We identified 142 down‐regulated microRNAs and 269 up‐regulated microRNAs that were differentially expressed between HNSC and normal tissues (fold change > 2, Figure S1A and B). We then used the GEO database (GSE28100) to further explored the abnormal expression of microRNAs in OSCC. A total of 22 up‐regulated microRNAs and 63 down‐regulated microRNAs were identified (fold change > 2, Figure S1C and D). We focused on the down‐regulated microRNAs in OSCC. The down‐regulated microRNAs identified in both of the two databases were has‐miR‐1258, has‐miR‐422a, has‐miR‐202 and has‐miR‐208a. We examined the expression level of these microRNAs in OSCC tissues and adjacent normal tissues. We revealed that has‐miR‐1258 decreased significantly in OSCC tissues, when compared with the other three microRNAs (Figure S1E). We thus chose has‐miR‐1258 for further study.

**Figure 1:**
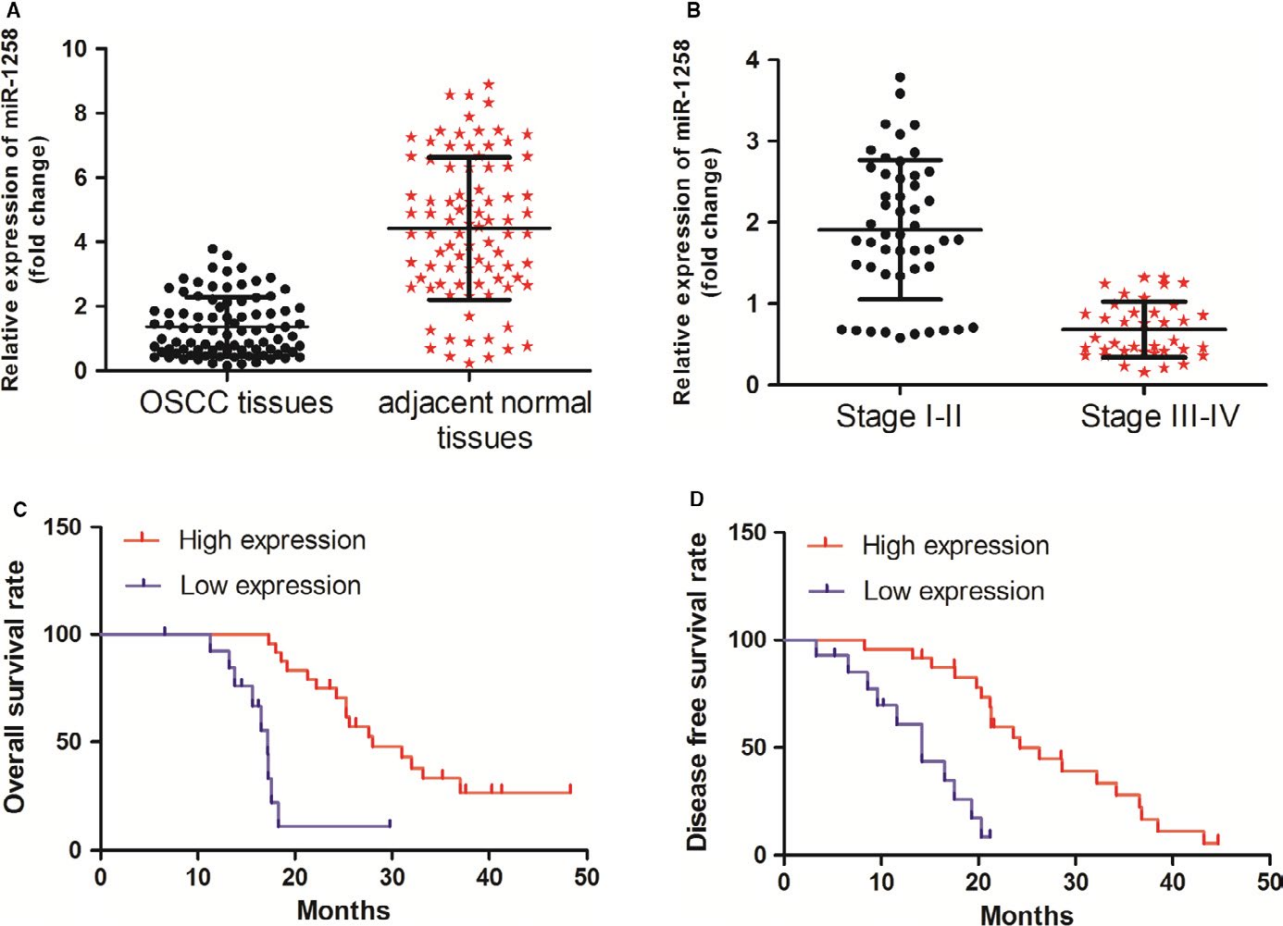
miR‐1258 expression was down‐regulated in oral squamous cell carcinoma (OSCC) tissues. (A) miR‐1258 expression was significantly decreased in OSCC tissues vs corresponding non‐tumour tissues, as determined by RT‐PCR. (B) miR‐1258 expression was negatively associated with OSCC clinical stage. (C) OSCC patients with lower levels of miR‐1258 had shorter overall survival when compared those with higher levels of miR‐1258. (D) Patients with higher miR‐1258 expression had better disease‐free survival rate than those with lower miR‐1258 expression

Subsequently, we examined the level of miR‐1258 expression in a larger cohort which contains 89 OSCC tissues samples (including the samples used before). It was confirmed that the expression of miR‐1258 was significantly down‐regulated in OSCC tissues when compared with that in adjacent normal tissues (Figure 1A, *P* < 0.05). Interestingly, we also revealed that the miR‐1258 expression was negatively associated with OSCC clinical stage (Figure 1B, *P* < 0.05).

### 3.2 Correlation of miR‐1258 expression with clinicopathological factors in OSCC

Subsequently, the correlation of miR‐1258 expression with clinicopathological factors in OSCC patients was evaluated. There was no significant difference between miR‐1258 expression level and gender, age and histological grade. However, we revealed that miR‐1258 down‐regulation was associated with T stage (*P* = 0.000), Lymphatic invasion metastasis = (*P* = 0.006) and distant metastasis (*P* = 0.009; Table 1). Next, we explored the relation between OSCC patients overall survival rate, disease‐free survival rate and miR‐1258 expression. It was found that OSCC patients with lower levels of miR‐1258 had shorter overall survival and disease‐free survival rate than those with higher levels (Figure 1C and D).

**Table 1:** Correlation between miR‐1258 expression and clinicopathological profiles

MiR‐1258 expression		Clinicopathological profiles	n	*P* value
High	Low	Gender		
27	23	Male	50	0.359
21	26	Female	47	
		Age		
25	20	<60	45	0.266
23	29	≥60	43	
		Histological grade		
23	25	Moderate + poor	46	0.760
25	24	Well	42	
		T stage		
34	16	T1‐2	49	0.000
14	33	T3‐4	39	
		Lymphatic invasion		
29	17	Negative	45	0.011
19	32	Positive	43	
		Distant metastasis		
12	24	Yes	36	0.015
36	25	No	52	

### 3.3 miR‐1258 directly targeted SP‐1 in OSCC cells

**Figure 2:**
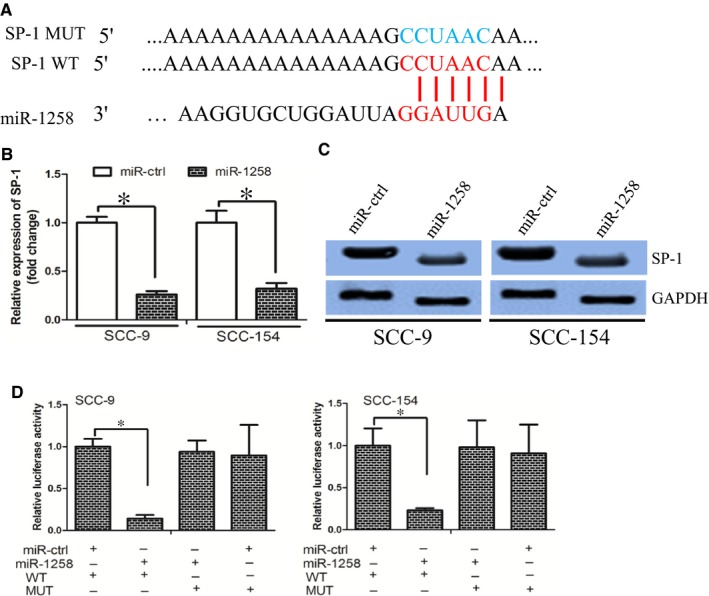
miR‐1258 directly targeted SP‐1. (A) SP‐1 wild‐type (WT) and mutant (MUT) 3′‐UTR as indicated. (B) and (C) miR‐1258 decreased SP‐1 expression at mRNA and protein level respectively. (D) miR‐1258 decreased the luciferase activity of SP‐1 WT 3′‐UTR instead of MUT 3′‐UTR in OSCC cells

MicroRNAs exert its function through targeting their targets and we searched the potential targets of miR‐1258 by TargetScan and miRanda. The SP‐1 protein was identified as a potential target of miR‐1258 (Figure 2A). The RT‐PCR and Western blot assay demonstrated that miR‐1258 inhibited SP‐1 mRNA and protein expression respectively (Figure 2B and C). We performed luciferase reporter assay to determine whether miR‐1258 directly targeted 3′‐UTR region of SP‐1. The 3′‐UTR region of SP‐1 mRNA including the predicted miR‐1258 recognition site (wild‐type) or the mutated sequence (mutant type) were subcloned into luciferase reporter plasmids (Figure 2A). We revealed that miR‐1258 decreased luciferase activity in the wild‐type vector, but not that in the mutant type vector (Figure 2D).

### 3.4 SP‐1 mediated miR‐1258’s effect on cell growth and invasion

**Figure 3:**
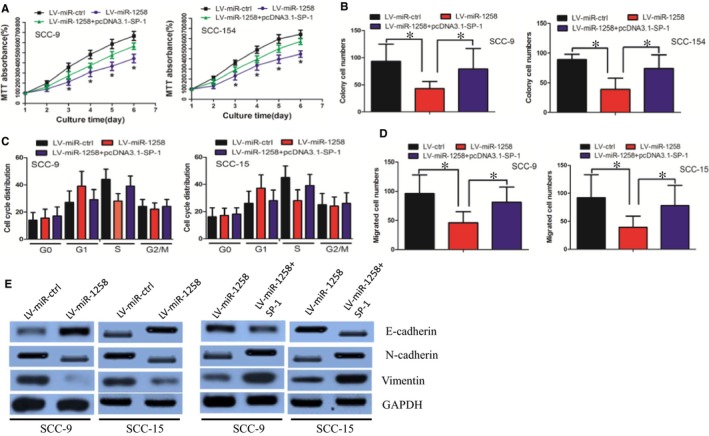
SP‐1 mediated miR‐1258’s effect on cell growth and invasion. (A) MiR‐1258 decreased oral squamous cell carcinoma (OSCC) cell growth, while overexpression of SP‐1 counteracted this effect, as determined by MTT assay. (B) MiR‐1258 impaired OSCC cell colony formation ability, while SP‐1 restoration counteracted the effect. (C) MiR‐1258 delayed cell cycle progression from the G1 phase to S phase, whereas this effect was dismissed by SP‐1 restoration. (D) MiR‐1258 decreased cell invasion ability, which was offset by SP‐1 overexpression. (E) MiR‐1258 inhibited the EMT phenotype, while the effect was neutralized by SP‐1 overexpression

First, we established OSCC cells stably expressing miR‐1258 by using lentiviral vector‐mediated overexpression (LV‐miR‐1258). Cells were also transduced with a control lentiviral vector (LV‐ctrl). The cell viability was decreased in LV‐miR‐1258 group when compared with that in LV‐ctrl group, as determined by the MTT assay (Figure 3A). In parallel, the LV‐miR‐1258 cells formed smaller and fewer colonies than the LV‐ctrl cells (Figure 3B). We then investigated whether miR‐1258 affected cell growth via altering cell cycle progression. We observed a lower proportion of S phase and a higher proportion in G1 phase in LV‐miR‐1258 cells compared with that in LV‐ctrl cells (Figure 3C). Our findings demonstrated that miR‐1258 inhibited OSCC cell growth by affecting cell cycle progression from the G1 phase to S phase.

Subsequently, we investigated whether miR‐1258 regulated cell invasion ability. We revealed that miR‐1258 decreased cell invasion ability, as determined by the Boyden assay (Figure 3D). We further explored whether miR‐1258 inhibited the EMT phenotype, which was responsible for cancer cell invasion. It was observed that the expression of the epithelial marker E‐cadherin increased, whereas expression of the mesenchymal markers N‐cadherin and Vimentin decreased in LV‐miR‐1258 cells, as determined by the Western blot assay (Figure 3E). In all, these data demonstrated that miR‐1258 inhibited EMT phenotype in the OSCC cells.

We also performed rescue experiment to determine whether miR‐1258 exerted its function mainly through SP‐1. It was revealed that overexpression of SP‐1 counteracted miR‐1258’s effect on cell growth, cell cycle distribution, invasion and EMT phenotype (Figure 3A‐E).

Taken together, our findings revealed that miR‐1258 decreased OSCC cell growth and invasion ability through regulating SP‐1 expression.

### 3.5 c‐Myb decreased miR‐1258 expression through binding at its promoter

**Figure 4:**
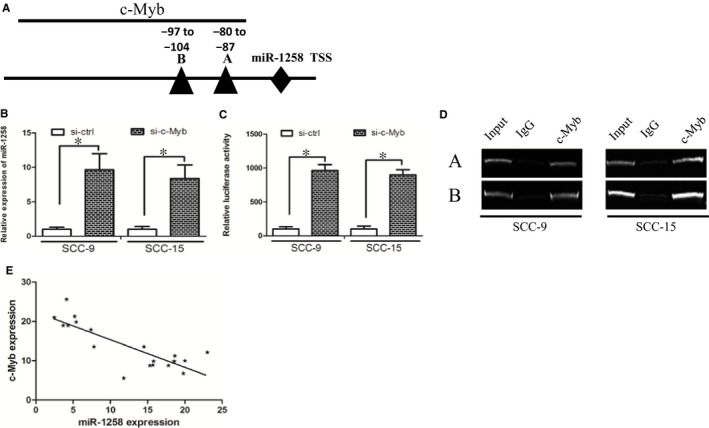
c‐Myb decreased miR‐1258 expression through binding at its promoter. (A) The putative transcription factor‐binding sites of c‐Myb at miR‐1258 promoter region. (B) c‐Myb down‐regulation increased miR‐1258 expression. (C) c‐Myb down‐regulation increased miR‐1258 promoter luciferase activity. (D) The chromatin immunoprecipitation assay confirmed that c‐Myb protein was recruited to all the four binding sites in the putative miR‐1258 promoter. (E) miR‐1258 expression was negatively correlated with c‐Myb expression in oral squamous cell carcinoma tissues

We used UCSC and PROMO bioinformatics software to analyse a 1‐kb region upstream of the transcription start site of miR‐1258. Two c‐Myb‐binding motifs at −80 to −87, and −97 to −104 were identified inside the putative promoter region upstream of the miR‐1258 transcriptional start site (TSS). We named these transcription factor‐binding sites (TFBSs) A and B (Figure 4A). Subsequently, we used si‐RNAs to knock down c‐Myb expression in OSCC cells and found that miR‐1258 expression was significantly increased in these cells when c‐Myb was down‐regulated (Figure 4B). In addition, c‐Myb down‐regulation increased miR‐1258 promoter luciferase activity (Figure 4C). Finally, the chromatin immunoprecipitation (ChIP) assay confirmed that c‐Myb protein was recruited to all the four binding sites in the putative miR‐1258 promoter in SCC‐15 and SCC‐9 cells (Figure 4D). We further revealed that miR‐1258 expression was negatively correlated with c‐Myb expression (Figure 4E, Spearman's correlation coefficient, *R* = −0.6635).

### 3.6 miR‐1258 inhibited PI3K/AKT and ERK pathways

**Figure 5:**
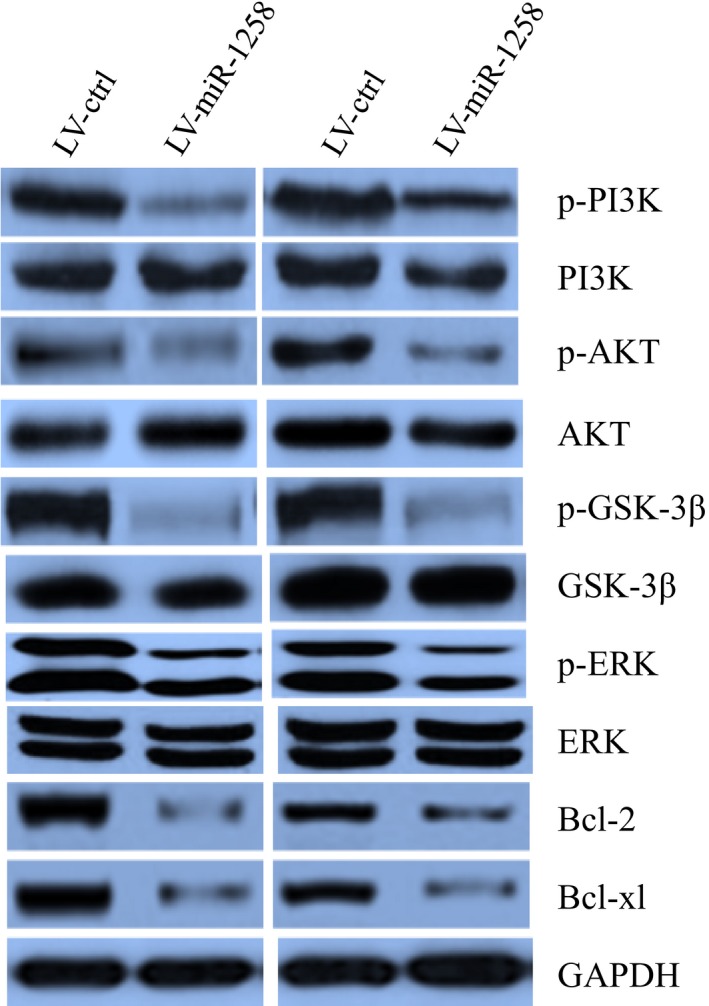
miR‐128 inhibited PI3K/AKT and ERK signalling pathway activity

The PI3K/AKT and ERK pathways were well‐known involved in cancer migration and proliferation, we thus investigated whether miR‐1258 regulated the PI3K/AKT and ERK pathways. It was revealed that miR‐1258 decreased level of phosphorylated PI3K, AKT, GSK‐3β and ERK, whereas total levels of these proteins remained unchanged. In addition, the expression levels of anti‐apoptosis proteins (bcl‐2, bcl‐xl) were decreased in miR‐1258 cells (Figure 5). Our data suggest that miR‐1258 decreased PI3K/AKT and ERK pathways.

### 3.7 Restoration of miR‐1258 decreased cell growth in vivo

**Figure 6:**
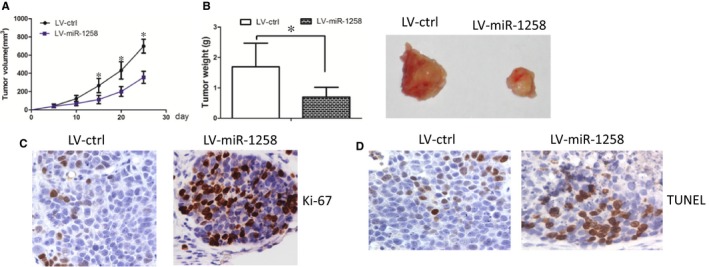
Restoration of miR‐1258 decreased cell growth in vivo*. *(A) LV‐miR‐1258 cell‐derived xenograft tumours grew more slowly when compared with the LV‐ctrl cell‐derived xenograft tumours. (B) The mean weight of LV‐miR‐1258 cell‐derived xenograft tumours was less when compared with LV‐ctrl cell‐derived xenograft tumours. (C) The Ki‐67 staining assay also revealed that LV‐miR‐1258 cells had less proliferation index than the LV‐ctrl cells. (D) The tunnel assay revealed that the apoptosis rate was increased in LV‐miR‐1258 cells, when compared with that in LV‐ctrl cells

We further asked whether miR‐1258 increased cell growth in vivo. LV‐miR‐1258 or LV‐ctrl cells were inoculated into the back of the nude mice respectively. Compared with LV‐ctrl cell‐derived xenograft tumours, LV‐miR‐1258 cell‐derived xenograft tumours grew more slowly when compared with the LV‐ctrl cell‐derived xenograft tumours (Figure 6A). In addition, the mean weight of LV‐miR‐1258 cell‐derived xenograft tumours was less when compared with LV‐ctrl cell‐derived xenograft tumours (Figure 6B). Interestingly, the Ki‐67 staining assay also revealed that LV‐miR‐1258 cells had less proliferation index than the LV‐ctrl cells (Figure 6C). The TUNEL assay revealed that miR‐1258 increased cell apoptosis in cell‐derived xenograft tumours (Figure 6D).Taken together, these results suggested that miR‐1258 decreased OSCC cells growth and increased cell apoptosis in vivo.

## 4 DISCUSSION

OSCC often develop cervical lymph node and distant organ metastasis because of their aggressive characteristics. Thus, it is urgent to elucidate the molecular mechanisms underlying OSCC tumourigenesis. MiRNAs have played significant roles in the invasion and metastases of malignant tumour cells. Currently, a list of oncogenic miRNAs is considered to be potential serological markers of OSCC.^12^ In breast cancer, miR‐1258 can target heparanase and subsequently inhibit cell aggressive phenotype.^8^ Similar results were observed in non‐small‐cell lung cancer and gastric cancer.^6^, ^7^ In addition, miR‐1258 was down‐regulated in hepatocellular carcinoma and inhibited liver cancer cell growth, proliferation and tumourigenicity through increasing cell cycle arrest in G0/G1 phase and promotes cell apoptosis.^13^ These data suggested that miR‐1258 functioned as tumour suppressor in cancer. However, the role of miR‐1258 in OSCC has seldom been investigated.

In the present study, we reported that miR‐1258 expression was down‐regulated in OSCC tissues. Subsequently, we uncovered that miR‐1258 was associated with T stage, lymphatic invasion metastasis and distant metastasis. Interestingly, the OSCC patients with higher levels of miR‐1258 had higher overall survival and disease‐free survival rate than those with lower levels. Consistent with previous studies, our data suggested that miR‐1258 may function as a tumour suppressor in OSCC.

In the functional experiment, we revealed that miR‐1258 overexpression decreased OSCC cell growth and invasion. The MTT and colony formation assays demonstrated that miR‐1258 decreased cell growth ability in OSCC. The cell‐cycle progression is a predominant factor promoting tumour cell growth. We observed that miR‐1258 delayed cell cycle from G1 phase to S phase and proposed that miR‐1258 may decrease OSCC cell growth ability through affecting cell cycle distribution. In parallel, the in vivo study revealed that miR‐1258 inhibited cell growth in the nude mice, which consolidated the data from the in vitro study.

Subsequently, we revealed that miR‐1258 decreased OSCC cell invasion ability by using Boyden assay. The EMT phenotype is tightly associated with cell invasion ability and we thus examined whether miR‐1258 inhibited EMT phenotype. The Western blot and immunofluorescence assays demonstrated that miR‐1258 increased E‐cadherin expression while decreased N‐cadherin and Vimentin expression. We speculated that miR‐1258 may inhibit EMT phenotype and ultimately decreased cell invasion.

Emerging evidence demonstrated the role of SP‐1 in cancer progression, invasion and metastasis.^14^ SP‐1 promoted cell proliferation through accelerating cell cycle from G1 to S phase.^15^ These data suggested that SP‐1 promoted cancer progression by affecting cell growth and invasion. A previous study confirmed that overexpression of SP‐1 contributed to OSCC development and targeting SP‐1 may be a potential therapeutic target in OSCC.^16^ Our findings revealed that miR‐1258 inhibited OSCC cell growth and invasion, whereas, restoration of SP‐1 counteracted miR‐1258’s effect. These findings suggested that SP‐1 may mediate miR‐1258’s function in OSCC.

Subsequently, we explored the underlying mechanism that contributed to the dysregulation of miR‐1258 in OSCC. Recruitment of specific transcription factors often led to abnormal miRNAs expression at genetic or epigenetic levels.^17^ The transcription factor c‐Myb is a key regulator of cell growth and metastasis in cancer. We revealed two putative biding sites of c‐Myb in the region upstream of miR‐1258 locus. The subsequent experiment demonstrated that c‐Myb could negatively regulate miR‐1258 expression by directly binding at its promoter. We further confirmed that there was a negative correlation between c‐Myb and miR‐1258 in the OSCC tissues. Taken together, our findings revealed that c‐Myb was responsible for miR‐1258 un‐regulation in OSCC.

Overall, our data provide the first evidence that the c‐Myb/miR‐1258/SP‐1 axis controls cell growth and invasion in OSCC cells. As down‐regulation of miR‐1258 is associated with poor prognoses and restoration of miR‐1258 decreased cell growth and invasion ability, therapeutics that target miR‐1258 may improve the treatment of OSCC.

## 5 CONCLUSIONS

In conclusion, our study demonstrated that miR‐1258 was down‐regulated in OSCC and may function as a tumour suppressor by targeting SP‐1, which consequently inhibited OSCC growth and metastasis. Our findings indicated that miR‐1258 could be a potential therapeutic target for OSCC treatment.

## ETHICS APPROVAL AND CONSENT TO PARTICIPATE

The study on OSCC cancer samples was approved and supervised by the Research Ethics Committee of Guangdong General Hospital, Guangdong Academy of Medical Sciences. Written Informed Consents were obtained from all patients. The animal experiments were performed in strict accordance with the guidelines of the Research Animal Care Committee Guangdong General Hospital.

## CONFLICT OF INTEREST

The authors declare that they have no competing interests.

## AUTHOR CONTRIBUTIONS

Study concept and design: Hua Zhang and Xiaoning Luo; acquisition of data: Xiaoning Luo; drafting of the manuscript: Hua Zhang. All authors read and approved the final manuscript.
