# Supplementary figures and images for: MicroRNA‐1258, regulated by c‐Myb, inhibits growth and epithelial‐to‐mesenchymal transition phenotype via targeting SP1 in oral squamous cell carcinoma
Source: J Cell Mol Med. 2019 Feb 7;23(4):2813–21. doi: 10.1111/jcmm.14189 (PMC6433684; doi:10.1111/jcmm.14189)

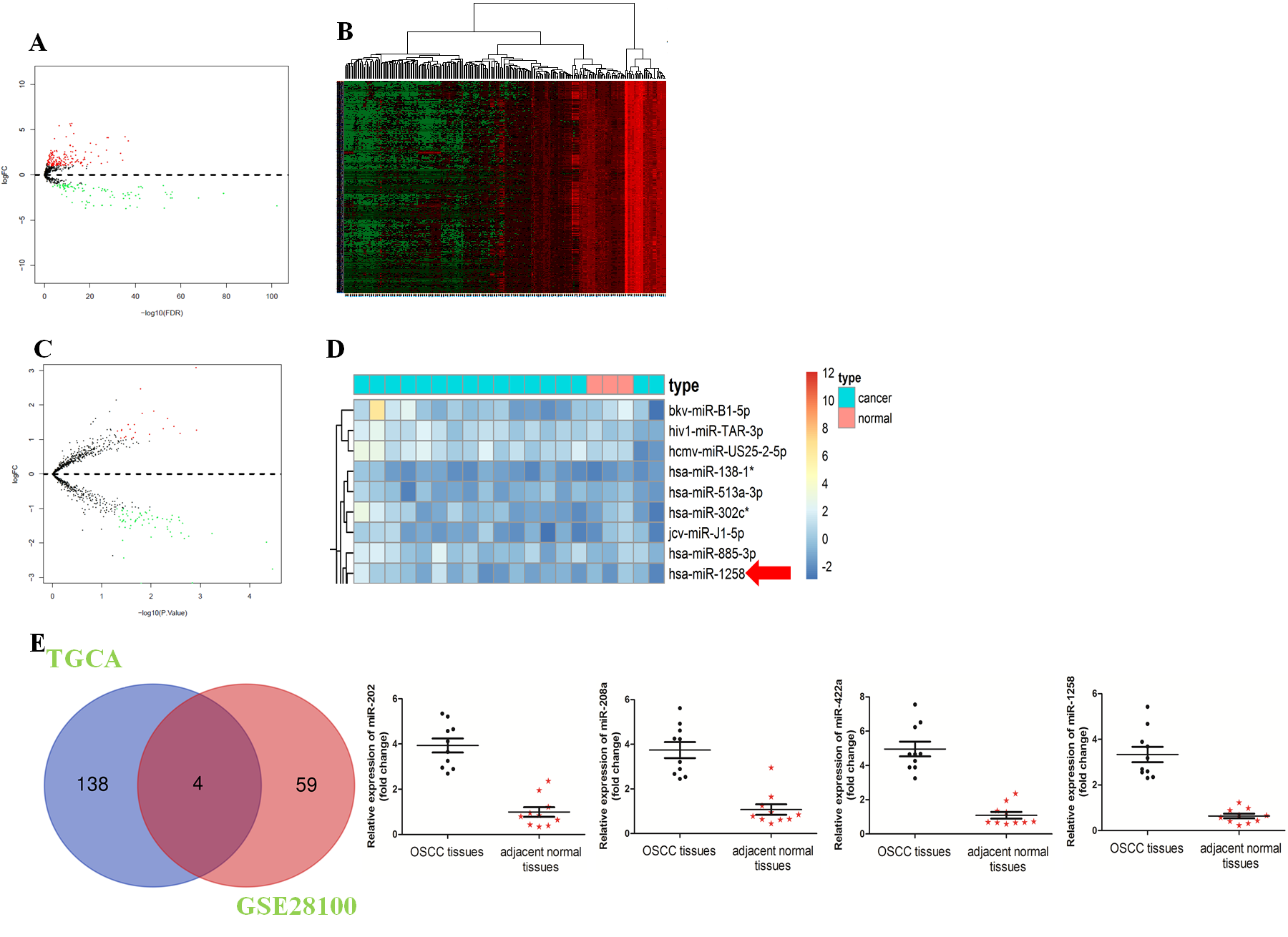

Supplement: Supplementary file 1 [file JCMM-23-2813-s001.tif]
